# Using Clustering Methods to Map the Experience Profiles of Dementia Caregivers

**DOI:** 10.1093/geroni/igae046

**Published:** 2024-05-18

**Authors:** Sato Ashida, Freda B Lynn, Lena Thompson, Laura M Koehly, Kristine N Williams, Maria S Donohoe

**Affiliations:** Department of Community and Behavioral Health, College of Public Health, University of Iowa, Iowa City, Iowa, USA; Department of Sociology and Criminology, College of Liberal Arts and Sciences, University of Iowa, Iowa City, Iowa, USA; Department of Community and Behavioral Health, College of Public Health, University of Iowa, Iowa City, Iowa, USA; Social and Behavioral Research Branch, National Human Genome Research Institute, Bethesda, Maryland, USA; School of Nursing, University of Kansas, Kansas City, Kansas, USA; Department of Community and Behavioral Health, College of Public Health, University of Iowa, Iowa City, Iowa, USA

**Keywords:** Caregiving networks, Dementia, Family caregiving, *k*-Means clustering

## Abstract

**Background and Objectives:**

Caregivers of persons living with dementia report wide-ranging lived experiences, including feelings of burden and frustration but also positivity about caregiving. This study applies clustering methodology to novel survey data to explore variation in caregiving experience profiles, which could then be used to design and target caregiver interventions aimed at improving caregiver well-being.

**Research Design and Methods:**

The *k*-means clustering algorithm partitioned a sample of 81 caregivers from the Midwest region of the United States on the basis of 8 variables capturing caregiver emotions, attitudes, knowledge, and network perceptions (*adversity*: burden, anxiety, network malfeasance; network nonfeasance; *positivity*: positive aspects of caregiving, preparedness and confidence in community-based care, knowledge about community services for older adults, and network uplift). The experience profile of each segment is described qualitatively and then regression methods were used to examine the association between (a) experience profiles and caregiver demographic characteristics and (b) experience profiles and study attrition.

**Results:**

The clustering algorithm identified 4 segments of caregivers with distinct experience profiles: *Thriving* (low adversity, high positivity); *Struggling with Network* (high network malfeasance); *Intensely Struggling* (high adversity, low positivity); *Detached* (unprepared, disconnected, but not anxious). Experience profiles were associated with significantly different demographic profiles and attrition rates.

**Discussion and Implications:**

How caregivers respond to support interventions may be contingent on caregivers’ experience profile. Research and practice should focus on identifying public health strategies tailored to fit caregiver experiences.

**Clinical Trial Registration:**

NCT03932812

**Translational Significance:** Because caregiving is not a one-size-fits-all experience, this study profiled the holistic experiences of families caring for persons living with dementia to ultimately improve the design of caregiver well-being interventions. Using multiple adversity and positivity measures related to caregiving experiences, we identified 4 distinct segments of caregivers: *Thriving, Struggling with Network, Intensely Struggling, and Detached*. These 4 experience profiles could help explain caregiver persistence in a recent study whereas none of the adversity and positivity measures alone could predict retention. Findings suggest developing interventions that are better suited to caregiver experiences may maximize impact and retention.

## Background and Objectives

Caregivers of persons living with dementia report wide-ranging lived experiences, including feelings of frustration and burden but also positivity through caregiving. On the one hand, caregivers experience challenges like performing complex medical care tasks, taking care of financial and legal aspects for the care recipient, and managing interpersonal interactions with healthcare providers and other family members (1). These care-related stressors are referred to as primary and secondary stressors in the Stress Process Model (2), a widely used model to understand caregiving experiences. Due to these stressors, caregivers often experience caregiving burden that leads to emotional and physical ill health as well as decreased levels of care provided (3).

At the same time, caregivers report positive experiences through caregiving (4). For example, some report positive aspects like enhanced personal strengths and competence, social relationships, perspectives on life, and opportunities to give back (5). Focusing on positive aspects of caregiving can act as a coping resource and help buffer stressors (6,7), ultimately benefiting caregiver health and well-being (8). Given that most caregivers experience both negative and positive aspects of caregiving, efforts to understand and improve caregiver experiences should conceptualize caregiving as a holistic experience considering both adversity and positivity.

A major element of this holistic experience is the social interactions associated with caregiving (9). Strong social networks are important in facilitating caregiver adaptation (1), impacting the well-being of both care recipients and their caregivers (10) thereby supporting the care recipients’ ability to continue living in the community (11). Personal social networks are also impacted by caregiving. To attend to changing needs, network members continuously adjust their roles and interactions. Such adjustments can result in secondary stressors (eg, conflicts among members) that can lead to feelings of resentment, guilt, and depression (12,13) or positive aspects of caregiving (eg, strengthening of their network) that can foster caregiver well-being (5). Families’ ability to adapt to changes and resolve conflicts was associated with continued support (14) and care provision (15).

A previous study (16) characterized 3 types of social interactions with respect to siblings caring for their older parent: malfeasance (“active negative behaviors” such as telling “others what to do” without helping), nonfeasance (others not engaged: “not doing a fair share” or “not showing interest.”), and uplift (supportive interactions). This study showed that nonfeasance and malfeasance were linked to higher strain among all siblings (16). In another study, network members perceived to engage in malfeasant or nonfeasant interactions and behaviors were perceived to be falling short of expectations, and a higher number of network members not meeting expectations was associated with caregivers reporting higher levels of depressive symptoms (17). Qualitative studies showed unmet expectations in caregiving (18) were perceived as most pervasive and stressful. In sum, interpersonal strain and support are both important dimensions of the caregiving experience.

### Holistic Caregiver Experiences: Creating Caregiving Experience Profiles

The research described earlier points to critical ways in which caregiving experiences can differ. Research on these critical differences generally takes a single-outcome approach. For example, many investigate the extent to which individuals report higher or lower levels of a specific variable, such as caregiving self-efficacy, and the extent to which this variation can be explained by known factors, such as level of social support (19). Another common question is asking the extent to which one variable, such as caregiver report of available social support, predicts a single-outcome variable, such as feelings of caregiver burden (20). In contrast, our study does not examine low-to-high variation in a single outcome but rather seeks to explore qualitative variation in holistic experiences; we do so by sorting the population into groups who share a similar *pattern* of experiences across multiple variables.

Building from Singer et al.’s (21) life-history method, we define a holistic experience as an individual’s array of experiences, including emotions, attitudes, and interpersonal mechanisms in the caregiving context. A person’s experience is a multidimensional configuration of variables—for instance, one individual could simultaneously feel (a) a great deal of burden and anxiety related to caregiving, (b) deeply unprepared for caregiving duties, and (c) entirely disappointed by their network contacts who are supposed to be helping. In contrast, a different individual might experience a great deal of burden and anxiety and yet feel prepared for their duties and report no network strife. These 2 experiences are holistically different, and highlight the question of whether interventions aimed at helping caregivers could be more effective if explicitly designed with population heterogeneity in mind rather than implicitly based on the assumption of homogeneity (22).

### Implications of a Holistic Approach for Caregiving Interventions

A holistic approach allows us to explore the possibility that there are distinct types of caregiving experiences. Clustering caregivers on their shared experiences can reveal that the caregiving population faces different types of challenges reflected in distinct experience profiles (see eg, Chow (23); Pruinelli et al. (24); Alhlqvist et al. (25)). In turn, these experience profiles can directly inform how interventions aimed at facilitating caregiver well-being are designed. For example, intervention strategies would differ for those who are experiencing high levels of negativity on many dimensions compared to those who are mostly experiencing positivity but struggle specifically with 1 type of caregiving-related burden. Likewise, a better understanding of caregiver experience profiles could help intervention designers identify and recruit those in the population who are most likely to benefit from a certain type of intervention.

Large and increasing numbers of individuals are diagnosed with Alzheimer’s disease and related dementias. About 80% live in the community (26) and are supported by informal caregivers (eg, family, friends) (27). Importantly, caregivers of persons living with dementia experience higher levels of depression, have a lower quality of life (28), and are at an increased risk of mortality (29) compared to caregivers of other older adults. Thus, there is an urgent need to develop effective intervention models (30); knowing more about population heterogeneity in caregiving experiences related to Alzheimer’s disease and related dementias is critical in designing interventions that address more specific caregiving challenges to increase effectiveness.

To this end, there are 3 aims for this paper. First, we use clustering techniques to divide a sample of caregivers into segments based on their shared experiences with adversity and positivity in the caregiver role. Clustering techniques have been employed in a diverse array of empirical contexts to study life and disease experiences (eg, Nnoaham and Cann (22); Chang et al. (31); Chow et al. (23)), but have yet to be applied to the caregiving experience specifically. Second, after describing the unique experience profiles of each segment, we then explore the demographic profile of each segment with respect to caregiver characteristics and functional levels of the person living with dementia as a proxy for the severity of symptoms. Doing so helps us understand if certain types of individuals are more likely to experience caregiving in a certain way. Third, we examine the extent to which the segments created by the clustering routine help us predict the extent to which caregivers persist in an intervention study for 6 months (regardless of control or intervention status). Although there are many reasons why a caregiver might stop participating in a program, this analysis allows us to examine if experience profile can systematically account for variation in dropout likelihood.

## Research Design and Methods

### Setting and Procedures

Data were collected between October 2019 and April 2021 through a randomized trial to assess the efficacy of a caregiver support program. Specifically, the Building a Bridge intervention (NCT03932812) focused on proactively engaging families to maximize successful role transitions by increasing knowledge and use of community-based services and enhancing social support. The Building a Bridge intervention was specifically designed to support caregivers in Midwestern U.S. urban and rural areas who had recently (ie, within the past 2 years) learned of their care partners’ dementia diagnosis to help them prepare for what they likely would experience as they moved forward in their caregiving journey. There were 2 components to the Building a Bridge intervention, Options Counseling (OC), and Health Education (HE). The intervention was delivered by trained Options Counselors from a local Area Agency on Aging who were trained through a formal program that includes person-centered care planning, counseling, and motivational interviewing. Families randomized to the intervention arm received an initial OC call that included a family assessment, counseling, emotional support provision, access assistance, development of independent-living plans, and educational needs assessment. The counselor conducted up to 5 monthly follow-up calls to update the caregiver’s care plans and deliver the HE component. The HE component was developed for this study and consists of 7 educational modules: dementia overview, anticipated changes and challenges, social relationships and daily life, interacting with providers, addressing future needs, dementia and driving, and elder mistreatment.

Due to the coronavirus disease 2019 (COVID-19) pandemic, participants were recruited by mailing letters to the families of individuals who had received a dementia diagnosis within the past 2 years and lived in the state of Iowa and were identified through the electronic health records at University of Iowa Hospitals and Clinics. Research staff contacted those who returned positive responses to screen and scheduled a baseline interview. Those who did not respond received up to 3 phone calls to assess their interests and those who indicated interest were enrolled. English-speaking adult family, friends, or partners of persons diagnosed with dementia within the past 2 years were eligible if the person did not live in a nursing home or another care facility; the intervention focused on community-living persons living with dementia. All participants provided verbal consent, completed baseline phone interviews, and then were randomized into intervention or control arm. Those in the intervention arm received the program within a few weeks of baseline with monthly calls for the following 3–5 months. Follow-up interviews occurred 3 and 6 months after the baseline (see Supplementary Material 1). Caregivers in the control arm were given an opportunity to receive the Building a Bridge intervention after their 6-month interview. Study procedures were approved by the Institutional Review Board at the University of Iowa.

For the present study, the baseline data from 81 caregivers was used in the cluster analysis (Aim 1). Similarly, baseline data were used to assess how clusters differed with respect to their demographic composition and the functional needs of the person living with dementia (Aim 2). Exploratory and not hypothesis driven, Aim 2 uses often-studied characteristics to learn more about the background and circumstances of caregivers in each cluster. The analysis of attrition (Aim 3) was based on whether respondents completed a follow-up interview at 6 months, regardless of whether they were in the treatment or control group.

### Measures

#### Eight aspects of caregiving experiences

This study focuses on 8 recognized dimensions of how caregivers experience their day to day in the caregiving role, all of which were discussed in the Background section. There are 4 adversity measures (caregiving burden, anxiety, network malfeasance, and network nonfeasance) and 4 positivity measures (positive aspects of caregiving, preparedness and confidence in community-based care, knowledge of community services, and network uplift). Table 1 describes each variable and Table 2, panel A, provides a descriptive summary of each variable. Exact question wording and details of variable construction are available in Supplementary Material 2.

**Table 1.** Eight Variables Used in Clustering Analysis

Variable	Description
A. Positivity measures	
Positive aspects of caregiving (PAC)	PAC is a 10-question battery (alpha = 0.83) modified from Tarlow et al.’s 9-item scale (32). PAC is the sum of all items measured on a 5-point scale, where high values indicate agreement with statements such as “Providing care and support to this individual made me feel more useful.”
Preparedness and confidence in community-based care	This variable is the mean of 12 items (alpha = 0.89) and measures the extent to which caregivers feel prepared for the caregiver role (8 items), such as meeting the physical and emotional needs and successfully finding organizations (33), and self-efficacy to use relevant support services (4 items) (34). High scores represent a high sense of preparedness and confidence.
Knowledge about community services	Knowledge is a 7-item scale assessing the extent to which the participant reports knowing about formal services available in their community related to the care of older adults (alpha = 0.89) (35). Each item is measured on a 5-point scale; the maximum score of 35 corresponds to participants with the highest self-rated knowledge.
Network uplift	Using a previously developed scale (17), respondents identified specific contacts from their total list of contacts who offer different forms of support (1 = Yes, 0 = No). A high score indicates the presence of network help, that is, many contacts whom are helpful. A low score corresponds to the limited or absence of network support, that is, respondent does not view their contacts as helpful or named very few contacts altogether.
B. Negativity measures	
Burden	Perceived burden is measured as the 12-item Zarit burden scale (36). This variable is the sum of all items (alpha = 0.88) measured on a 5-point scale, where high values indicate that the respondent feels a great deal of burden associated with caregiving activity.
General anxiety	We use the 7-item General Anxiety Disorder (GAD) scale (37) to measure general nervousness, fear, and worry (alpha = 0.86). Each item is measured on a 4-point scale; the theoretical maximum is 21 for those experiencing the highest levels of anxiety.
Network malfeasance	Malfeasance refers to the extent to which respondents report frustration or negative emotion with their named contacts, such as perceptions of shirking or conflict over caregiving. Using a scale previously developed (18), respondents identified contacts who fits descriptions (1 = Yes, 0 = No). A high score indicates a higher degree of tension with contacts whereas a low score indicates the limited or absence of stress, which could indicate that the respondent does not perceive tension or has very few contacts altogether.
Network nonfeasance	Nonfeasance refers to the extent to which respondents report contacts not engaging in caregiving-related tasks. Using a scale previously developed (18), respondents identified contacts who engage in social interactions identified as nonfeasant (1 = Yes, 0 = No). A high nonfeasance score indicates that the participant feels that their caregiving network is not living up to expectations.

**Table 2.** Descriptive Summary of Variables

Variable	*n*	Mean	*SD*	%	Min	Max
A. Experience variables for cluster analysis						
Positive aspects of caregiving	81	33.72	7.90		12	50
Preparedness and confidence	81	2.18	0.70		0.37	3.83
Knowledge	81	23.11	7.73		7	35
Network uplift	81	34.47	27.54		0	128
Zarit burden	81	18.77	8.92		5	39
General anxiety	81	5.58	4.47		0	19
Network malfeasance	81	3.28	4.06		0	20
Network nonfeasance	81	5.19	6.97		0	32
B. Characteristics of caregivers and their person living with dementia						
Age (years)	81	67.51	13.51		26	86
Female	81			65%	0	1
% >$80K Income	77			40%	0	1
% College or advanced education degrees	81			52%	0	1
% Rural zip code	81			64%	0	1
% Spouse	81			73%	0	1
Percent lives with person living with dementia	81			85%	0	1
Length of care	81	3.90	0.87		2	5
1 = Less than 30 days				0%		
2 = 1–6 months				2%		
3 = 6 months to less than 2 years				36%		
4 = 2 years to less than 5 years				31%		
5 = More than 5 years				31%		
Quantity of care:	77	3.03	1.15		1	4
1 = Up to 8 h/wk				14%		
2 = 9 to 19 h/wk				21%		
3 = 20–39 h/wk				13%		
4 = 40 hours or more				52%		
FAST Score	81	3.10	2.26		0	9
Network size	81	11.36	6.05		2	26

*Notes*: All variables above are measured at the baseline interview. FAST = functional assessment staging; *SD* = standard deviation.

#### Caregiver and care recipient demographic measures

Table 2, panel B, summarizes 11 variables related to the characteristics of caregivers and persons living with dementia relevant to Aim 2, all of which are self-reported by the caregiver. The caregiver’s age is measured in years and the variable Female captures if the caregiver self-identified as female as opposed to male. No respondents identified as “other” with respect to their gender. Income is measured with respect to “total annual household income before taxes” and corresponds to a 5-category ordinal scale (1 = Under $20 000; 2 = $20 000–$39 999; 3 = $40 000–$59 999; 4 = $60 000–$79 999; and 5 = $80 000 or more). For simplicity, in Aim 2, we dichotomized the distribution at the 75th percentile (1 ≥$80 000; 0 = less). Similarly, caregiver’s highest level of education is measured with a 5-category ordinal scale (1 = Less than high school; 2 = High school; 3 = Post high school or Associate degree; 4 = 4-year college degree; 5 = Master’s/doctorate degree) that we then dichotomized based on a 4-year degree attainment (1 = attained a 4-year degree or more; 0 = less). Rurality is measured by zip codes that were coded using the Rural-Urban Community Area Codes (RUCA) (38) system (1 = RUCA codes 4–10 indicating rural; 0 = RUCA codes 1–3 indicating urban).

The caregiver’s relationship to the person living with dementia is coded as spouse (1) or nonspouse (0) based on an open-ended question (“What is your relationship to the individual who received the diagnosis?). The majority of caregivers are spouses (73%) with “child” or “child-in-law” as the next most common response (15%). Other responses are parent, sibling, grandchild, niece, and unrelated. The living arrangement is measured as a binary: “Does the individual who received a diagnosis of Alzheimer’s disease or other dementia live with you?” (yes=1; no=0). Two aspects of time invested in caregiving are measured with ordinal scales (see Table 2 for details): length of care (1 = Less than 30 days; 5 = more than 5 years) and quantity of caregiving (1 = Up to 8 hours per week; 4 = 40 hours or more). The Functional Assessment Staging (FAST) tool is used to assess the functional levels of persons living with dementia (39). Network size is computed from the network generator question described in the Network Uplift portion of the Supplement.

#### Study attrition

We measure attrition with respect to participation (yes vs no) at the 6-month follow-up interview. Of the original 81 baseline participants, 30 did not participate, yielding an overall attrition rate of 37%. Of the 30 who left the study, 4 participants in intervention and 1 in control group reported losing their care partner during the study. The 4 in intervention continued to receive support from the OC but chose not to complete the final survey. In the control arm, no systematic data are available (by definition) on why participants dropped out; participants who stopped returning calls could have done so because their care partner had died, or because they no longer wanted to participate for other reasons.

### Analysis

To address our first aim, we divide our sample of 81 caregivers based on similarity across the 8 adversity and positivity variables described above using the *k*-means clustering algorithm (40) available in Stata17. All variables were standardized prior to running the clustering routine. This unsupervised machine-learning method is iterative and begins by picking *k* centroids (ie, *k* caregivers and their 8 responses) at random to “anchor” a cluster, where *k* is specified by the analyst based on a combination of model-fit statistics and perceived parsimony and interpretability (41). Next, each remaining caregiver is assigned to one of *k* clusters based on its Euclidian distance from each centroid. The centroid for each cluster is then re-calculated once all caregivers have been assigned. These last 2 steps are then repeated until stable clusters are produced (all caregivers remain in the same group from the previous iteration). The best partition is the one that minimizes the total within-cluster distance between each data point and centroid. Each cluster or segment corresponds to a group of caregivers who experienced a similar pattern of adversity and positivity, which we refer to as a segment’s experience profile.

To briefly illustrate how this method works, imagine a data set with 100 respondents and 3 variables related to their frequency of different types of exercise (running, yoga, and tennis). The *k*-means clustering routine would divide the entire pool of respondents into a prespecified number of segments based on similarity in respondents’ exercise patterns, wherein each respondent belongs to 1 segment only. If the analyst asked for a 4-segment solution, the algorithm might reveal a segment of 40 respondents who *on average* run “frequently” but “never” practice yoga or play tennis; a segment of 20 who tend to do tennis and yoga “occasionally” but “never” run; a segment of 30 who generally “never” do any of the three; and a segment of 10 respondents who “frequently” do all three.

The next step after identifying segments is to create demographic profiles of each segment (Aim 2). Specifically, we examine if segments are systematically associated with certain demographic characteristics of caregivers or the severity of symptoms of the person living with dementia. We present both a descriptive summary of results and use ordinary least squares (OLS) and logit regression models to test if there are differences in means across segments. Aim 3 examines attrition at 6 months using logit regression models to detect if the likelihood of attrition differs by segment.

## Results

### Aim 1. Describing the Experience Profile of Each Caregiver Segment

**Figure 1.**
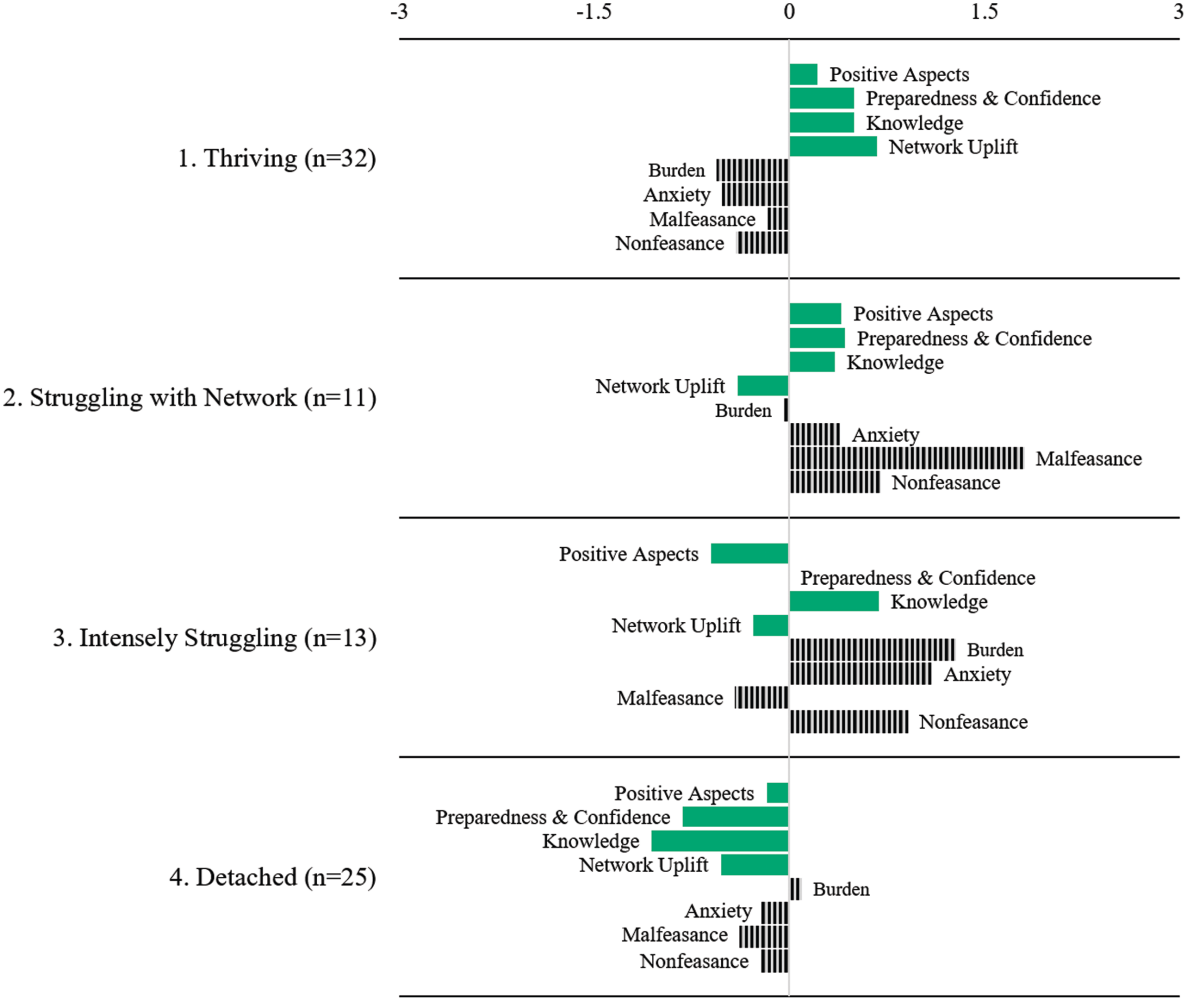
Caregiver experience profiles by segment. Striped bars refer to adversity measures and solid bars refer to positivity measures.

Based on substantive interpretability and model-fit statistics (42), such as the Calinski–Harabasz pseudo-*F* index and the Duda–Hart pseudo-*T*^2^ statistic, we divided the sample of 81 caregivers into 4 segments. Figure 1 summarizes the mean levels of each of the adversity and positivity variables by segment. Because all variables were standardized prior to clustering, a score of 0.0 indicates an average response relative to the full sample. Negative and positive scores thus indicate the extent to which caregivers within a segment tend to exhibit unusually low or high values on a particular variable. Striped bars refer to adversity variables and solid bars refer to positivity variables.

The first segment (*n* = 32) consists of caregivers who are *Thriving*. On average, these caregivers report a low level of burden and see many positives about the caregiving experience. They feel prepared and confident in their ability to navigate community resources and provide care to the person living with dementia, and they are low in general anxiety. This group characterizes their caregiving network as being low on malfeasance and nonfeasance and high on helpfulness, or uplift. The experience profiles of the remaining 3 segments are distinct. Caregivers who are *Struggling with Network* (*n* = 11), *Intensely Struggling* (*n* = 13), and *Detached* (*n* = 25) are each struggling in a unique way compared to those who are *Thriving*.

Those *Struggling with Network*, similar to *Thriving*, are well above the mean in terms of their positivity, preparedness and confidence, and knowledge about community services. Their anxiety, however, is high. The network measures suggest that this could be due to a lack of satisfactory network resources; specifically, those in this segment have caregiving networks characterized by above-average levels of malfeasance and nonfeasance and an absence of uplift or helpfulness.

The *Intensely Struggling* experience profile is similar to *Struggling with Network* insofar as both report network woes such as high nonfeasance and low uplift. That said, *Intensely Struggling* caregivers report much lower positivity and much higher levels of burden and anxiety compared to *Struggling with Network*, suggesting that those *Intensely Struggling* are far less likely to derive energy and purpose from caregiving. Another key difference is that *Intensely Struggling* caregivers exhibit low levels of uplift and high levels of nonfeasance without high levels of malfeasance, whereas *Struggling with Network* experiences all three. In other words, for *Intensely Struggling*, malfeasance and nonfeasance are not co-present even though they are positively correlated (*r* = 0.43) in the entire sample. Overall, the *Intensely Struggling* segment appears to be dealing primarily with a nonengagement problem whereas the *Struggling with Network* segment is dealing with a combination of nonengagement and conflict.

Finally, the *Detached* experience is unique in that those caregivers are below average on all negativity and positivity dimensions except for burden, for which they are average. As a segment, *Detached* is notably low with respect to preparedness and knowledge, which may reflect disengagement or overwhelm. In addition, caregiving networks for *Detached* are unusual compared to the other experience profiles in that they simultaneously lack support, malfeasance, and nonfeasance, suggesting relatively “empty” social networks in the context of caregiving.

### Aim 2. Describing the Demographic Background and Circumstance Profile of Each Segment

Table 2, panel B, provides the demographic characteristics of caregivers and persons living with dementia for the entire sample of 81 caregivers included in the cluster analysis. The average age was 67.5 years, ranging from 26 to 86 years, 65% were female, about half had a high level of education (post-secondary degrees), 98% self-identified as White, and 64% lived in a “rural” zip code based on the RUCA indicator (38). The majority (85%) lived with their person living with dementia; 47 (58%) of them were in the “mild” category of functional stage as determined by the FAST Scale (39). Another 30 (37%) were in the “middle” category, and 4 (5%) were categorized as “late” stage of dementia. Table 2 also shows that, with respect to length of care, some caregivers do report providing care for longer than 2 years. This is consistent with studies documenting that caregiving often starts before individuals receive a formal diagnosis (43).

Table 3 breaks down Table 2 by experience profile. Table 3 shows that there are some noteworthy patterns in terms of how experience profiles differ by the background characteristics of caregivers, which helps contextualize the findings in Figure 1. First, the composition of *Struggling with Network* is significantly different from *Thriving* with respect to caregiver age, spousal status, and length of care. We find that caregivers are statistically younger in *Struggling with Network* compared to *Thriving; Struggling with Network* is also a younger group of caregivers compared to both *Intensely Struggling* and *Detached*. *Struggling with Network* is also simultaneously the least likely to be a spouse compared to *Thriving* and their length of care is also statistically greater than *Thriving.* From a holistic and qualitative standpoint, *Struggling with Network* appears to be a more common experience for caregivers of persons living with dementia who are less educated and younger nonspousal female relatives.

**Table 3.** Caregiver Background and Circumstance Profiles by Segment

Variable	1. Thriving (*n* = 32)	2. Struggling With Network (*n* = 11)	3. Intensely Struggling (*n* = 13)	4. Detached (*n* = 25)	Total Sample
Age, mean (*SD*)	69.7 (11.4)	57.0**^,^^†^ (20.0)	70.4 (6.1)	67.8 (13.9)	67.5 (13.5)
Female	66%	91%	54%	60%	65%
% >$80K	43%	30%	38%	42%	40%
% College or advanced degree	50%	36%	85%*^,^^‡^	44%	52%
% Rural	69%	82%	54%	56%	64%
% Spouse	81%	36%**^,^^§^	92%	68%	73%
Respondent lives with person living with dementia (% Yes)	81%	82%	100%	84%	85%
FAST Severity Score of person living with dementia, mean (*SD*)	3.2 (2.3)	3.1 (2.4)	4.2 (2.2)	2.4^||^ (2.0)	3.1 (2.3)
Length of care, mean (*SD*)	3.7 (0.9)	4.4^*^ (0.9)	4.1 (0.9)	3.8 (0.8)	3.9 (0.9)
Hours of care, mean (*SD*)	2.8 (1.2)	2.7 (1.3)	3.5^**†**^ (0.7)	3.1 (1.2)	3.0 (1.1)
Caregiver network size, mean (*SD*)	13.4 (6.1)	12.0 (6.2)	11.7 (6.0)	8.3** (4.8)	11.4 (6.0)

*Notes*: *SD* = standard deviation. Superscripts indicate if group mean or proportion is significantly different from *Thriving* (two-tailed: ^†^*p* < .10, *p < .05, **p < .01).

^†^Also significantly different from *Intensely Struggling* (*p* < .05) and *Detached* (*p* < .05).

^‡^Also significantly different from *Struggling with Network* (*p* < .05).

^§^Also significantly different from *Intensely Struggling* (*p* < .05).

^||^Significantly different from *Intensely Struggling* (*p* < .05).

Second, *Intensely Struggling* devotes statistically more hours to caregiving compared to *Thriving,* which could be a reason for why those caregivers are struggling with anxiety, burden, low positivity, and network nonfeasance. *Intensely Struggling* also has a statistically higher percentage of members with a college or graduate degree compared to *Thriving*. Focusing just on graduate degrees, 54% of *Intensely Struggling* reports having some type of advanced degree compared to only 31% of *Thriving*, 18% of *Struggling with Network*, and 16% of *Detached*. Overall, we observe that all caregivers in *Intensely Struggling* live with the person living with dementia and that this segment consists of the highest proportion of husbands (46%) of the 4 segments. More specifically, *Intensively Struggling* has the highest proportion of husbands with graduate degrees (31%). In comparison, well-educated husbands make up only 13% of *Thriving*, 0% of *Struggling with Network*, and 4% of *Intensely Struggling*.

Finally, *Detached* is demographically comparable to *Thriving* with the key exception that *Detached* caregivers report significantly smaller caregiving networks compared to those in *Thriving*. This finding helps contextualize their experience profile, which we showed above was low in network help and network tension. *Detached* caregivers are also caring for persons with the lowest FAST scores of the sample, but statistically symptom severity is the same as *Thriving*. Taken all together, there is no evidence that *Detached* caregivers are newer to caregiving compared to *Thriving* and yet those in *Detached* have severely underdeveloped network resources compared to *Thriving*.

### Aim 3. Using Cluster Results to Predict Research Study Attrition

The fitted logit model showed that the 22% of caregivers in *Thriving* (7 of 32) who did not complete the 6-month follow-up interview is statistically lower than the 55% (6 of 11) who dropped out in *Struggling with Network* (*p* = .050) and the 56% (14 of 25) who dropped out in *Detached* (*p* = .010). The attrition rate for *Intensely Struggling* is also relatively low (23%, 3 of 13), which is statistically the same as the attrition rate for *Thriving.* In sum, the experience profiles are predictive of attrition over the 6-month period; *Struggling with Network* and *Detached* are roughly twice as likely to drop out compared to *Thriving* and *Intensely Struggling*. Results are substantively the same after removing the 5 participants whose care partner died during the study (results available upon request). In addition, we find that none of the 8 adversity and positivity variables on which the clustering analysis is based can significantly predict dropout when modeled as a single predictor (see Supplementary Material 3). For example, burden alone cannot predict attrition, a result that could be anticipated from Figure 1. Burden is unusually low in *Thriving* and unusually high in *Intensely Struggling*, and yet both segments have low attrition compared to *Struggling with Network* and *Detached*.

## Discussion and Implications

Study findings suggest that there are holistic differences in how caregivers from this pool were experiencing their role as caregivers. Of the 4 distinct experience profiles identified, the largest segment of caregivers (40% of the sample) appears to be *Thriving*; they feel prepared and confident in their caretaking duties, see the positivity in their situations, can effectively manage their anxiety and burden, and have cultivated a robust network of help that is comparatively high on uplift and low on tension. Those in the 3 remaining segments, however, are struggling far more compared to the *Thriving* group, but for reasons that are qualitatively distinct.

The point of these results is not to challenge or test existing theories about caregiving experiences but rather to highlight how elements of the caregiving experience previously identified by past research combine in non-additive ways to create distinct experience profiles. Some aspects of these newly identified clusters are not surprising given past research. For example, those who experience high burden and anxiety report longer hours spent on caregiving and lower levels of social support (44) and more conflicts with their social network members (17,18). But others could not have been anticipated, such as those intensely struggling having higher levels of education than those who are thriving; higher levels of education may normally be considered as a protective factor (45). Most notably, caregivers in *Thriving* and *Detached* are demographically comparable, and both present low levels of anxiety and network negativity. Yet their respective overall experiences with caregiving are notably distinct. Those in *Detached* may be at higher risk for long-term detriments because of lower levels of preparedness, knowledge about services, and network support and they were indeed more likely to drop out of the study earlier than those in *Thriving*. We do not believe such differences in caregiving experiences have been elucidated in prior literature, and this highlights an important area of future inquiry to inform primary prevention of caregiver distress.

### Implications for Caregiver Interventions

This study has 3 main implications for health professionals designing intervention strategies to fit caregiver needs and improve impacts. First, results showed that caregivers in *Thriving* and *Intensely Struggling* were less likely to drop out of the study compared to the other 2 segments. Notably, none of the underlying 8 variables defining the experience profiles could alone predict attrition, demonstrating the value of using these holistic experience profiles to understand caregiver needs. This finding can help identify strategies to lower attrition rates among the subsets of the population who are the least likely to persist, possibly by allocating more resources or varying the resources needed to support them. Given the knowledge about the 2 experience profiles with the lowest persistence rates (*Struggling with Network* and *Detached*), future research may focus on identifying strategies to support caregivers who are struggling with their support networks or who are seemingly detached from caregiving situations and building support networks.

Second, many of the caregivers who participated in this intervention study were *Thriving* at the baseline interview, and this group had a relatively high persistence rate in both control and treatment arms through the 6-month follow-up. A critical question for future research is whether those in *Thriving* at baseline were still in fact thriving at a later point in their caregiving journeys. That is, there may be some degree of erosion in caregiver well-being due to disease progression (46). At the same time, it is possible that those in *Thriving* have a resource foundation (network, psychological, financial) that generates continued positivity throughout the caregiving journey (47). If the latter is true, studies aimed at identifying the origins of the *Thriving* experience could potentially help inform intervention strategies for those who are not thriving.

Third, this research strongly suggests that caregiver struggles are not one-size-fits-all. More research is needed to confirm the extent to which the 4 profiles detected here are generalizable to broader samples (see Limitations), but the findings from this study point to a number of promising directions for future interventions. For example, caregivers in *Struggling with Network* are grappling specifically with low social support, high nonfeasance, and high social conflict (malfeasance) while those in *Intensely Struggling* are also struggling with low social support and high nonfeasance. Together, this strongly suggests that in the population of caregivers for persons living with dementia, there is likely a nontrivial proportion of caregivers who could benefit substantially from interventions designed to help caregivers and families to communicate caregiving needs, build social support systems, and manage caregiving-network social strain. Yet nonpharmacological interventions for family caregivers of community-dwelling older adults living with dementia continue to mostly address psychological factors through therapy, facilitating mindfulness, psychoeducation, and behavioral therapy (48). We urge future research to identify strategies for rewiring existing social relationships (49) that caregivers could then employ to enhance supportiveness and reduce malfeasance and nonfeasance in their caregiving network system.

Another insight, this time gleaned from the *Intensely Struggling* profile, is that certain caregivers may be struggling with caregiving not because they are under-resourced per se but because caregiving is experienced as particularly disruptive. For example, caregivers in *Intensely Struggling*, relative to *Thriving*, tend to spend more hours providing care. Thus, adding intervention components on how to provide specific caregiving tasks such as helping with activities of daily living often used in traditional caregiver programs for more advanced dementia caregiving (10) could be particularly impactful. *Intensely Struggling* consists of the highest proportion of well-educated husbands who are taking care of their spouse at home; they self-report high knowledge about how to activate resources for their persons living with dementia but are lowest by far on positive aspects of caregiving. Perhaps they are struggling because they are trying to balance caregiving with time-intensive professional careers and as a result, may have limited time to manage their support systems (50) and cope with their spouse’s dementia diagnosis.

Similarly, the *Detached* group suggests that public health professionals may want to consider “jumpstart” strategies among caregivers who seem lost in their caregiving journey. *Detached* are notably unprepared and unknowledgable relative to all other profiles, even though there is no statistical evidence that *Detached* is caring for those in earlier stages of disease progression. In fact, 3 out of the 5 caregivers who lost the person they were caring for during the study were in the *Detached* segment. Moreover, the networks of those in *Detached* are basically empty—notably smaller, inactive, or disconnected—compared to all other profiles. Efforts to support caregivers with this experience profile may need to focus on jumpstarting their motivation to plan for future caregiving and highlight the importance of developing and activating their support systems.

### Limitations

The key limitation of this analysis is sample size and representativeness. With 81 participants drawn from a pool of eligible caregivers in Midwestern U.S. communities, the sample as a whole is not nationally representative and the number of caregivers in each segment is relatively small, which limits confidence in statistical generalizability and the ability to robustly test if the demographic profiles of each segment are distinct. For example, 82% of *Struggling with Network* (*n* = 11) is from a rural zip code compared to only 54% of Intensely Struggling (*n* = 13), but this nearly 30-point gap is not statistically significant at the .05 level given the small size of each segment. We were also not able to investigate potential differences by race given that nearly every participant self-identified as white. More generally, our analysis of caregivers’ background characteristics was limited to variables regularly included in analyses of the caregiving experiences and the operationalization of each variable was guided by original survey wording. Future studies need to reach more caregivers from diverse social and cultural backgrounds and develop more theories as to why the demographic background of caregiver and symptom severity comes to shape caregiver holistic experiences. In addition, because of the significant impacts of the COVID-19 pandemic which took place during this study, our attrition rate findings also may be difficult to generalize to future samples.

Taken together, future studies are needed to investigate if our findings about caregiving experience profiles and their demographic composition hold across other data sources. If they do, the results would strongly suggest that intervention designers need to systematically consider caregiver profiles when developing programs and recruiting participants. Also, further research would be needed to explore how practitioners could identify in real time the profile that best characterizes a caregiver’s situation. In terms of identifying the profile that a respondent most closely resembles, we caution that with the *k*-means clustering method, all respondents get assigned to 1 segment, which means that some respondents clearly fit within their cluster (eg, those who fit the prototype closely) while others are on the outskirts and may only weakly resemble the prototype. There are other clustering methods, such as fuzzy clustering (51) that would give respondents a score for how closely they fit into a segment.

In addition, we do not know what happened in participants’ experience prior to the baseline interview that could have contributed to why caregivers felt the way they did at the baseline interview. For example, perhaps on average, the symptoms of the persons living with dementia in *Intensely Struggling* declined much faster than that of those in *Thriving*, which could then explain why their sense of positivity is so much lower. This, however, is one of the first studies to focus specifically on caregivers within 2 years of dementia diagnosis, helping us understand how caregiving experiences can vary in the stage directly after individuals receive a diagnosis.

## Conclusion

This study reveals major holistic differences in caregiver experiences in the context of families caring for persons living with dementia in Midwestern U.S. communities. These holistic differences give rise to 3 recommendations for those researching and designing caregiver interventions: (a) develop caregiver support strategies tailored to experience profiles and test if they improve retention and positive impact; (b) refine theory and build empirical knowledge around the resource foundation that underlies the *Thriving* experience; and (c) identify strategies for rewiring and strengthening existing social relationships to enhance supportiveness and reduce adversity in caregiving network systems.

## Supplementary Material

igae046_suppl_Supplementary_Materials

## Data Availability

This study was preregistered (NCT03932812). Data and materials will be made available upon request by contacting the first author.

## References

[1] Pearlin LLI , Mullan JJT, Semple SSJ, Skaff MMM. Caregiving and the stress process: an overview of concepts and their measures. Gerontologist. 1990;30(5):583–594. 10.1093/geront/30.5.583 2276631

[2] Lazarus RS , Folkman S. Stress, Appraisal, and Coping. Springer; 1984.

[3] Liu Z , Heffernan C, Tan J. Caregiver burden: a concept analysis. Int J Nurs Sci. 2020;7(4):438–445. 10.1016/j.ijnss.2020.07.012 33195757 PMC7644552

[4] Lawton MP , Kleban MH, Moss M, Rovine M, Glicksman A. Measuring caregiving appraisal. J Gerontol. 1989;44(3):P61–P71. 10.1093/geronj/44.3.p61 2715587

[5] Peacock S , Forbes D, Markle-Reid M, et al. The positive aspects of the caregiving journey with dementia: using a strengths-based perspective to reveal opportunities. J Appl Geronto. 2010;29(5):640–659. 10.1177/0733464809341471

[6] Carbonneau H , Caron C, Desrosiers J. Development of a conceptual framework of positive aspects of caregiving in dementia. Dementia. 2010;9(3):327–353. 10.1177/1471301210375316

[7] Kramer BJ. Gain in the caregiving experience: where are we? What next? Gerontologist. 1997;37(2):218–232. 10.1093/geront/37.2.218 9127978

[8] Liew TM , Luo N, Ng WY, Chionh HL, Goh J, Yap P. Predicting gains in dementia caregiving. Dement Geriatr Cogn Disord. 2010;29(2):115–122. 10.1159/000275569 20150732

[9] Koehly LM , Ashida S, Schafer EJ, Ludden A. Caregiving networks-using a network approach to identify missed opportunities. J Gerontol B Psychol Sci Soc Sci. 2015;70(1):143–154. 10.1093/geronb/gbu111 25224254 PMC4296206

[10] Gitlin LN , Belle SH, Burgio LD, et al.; REACH Investigators. Effect of multicomponent interventions on caregiver burden and depression: the REACH multisite initiative at 6-month follow-up. Psychol Aging. 2003;18(3):361–374. 10.1037/0882-7974.18.3.361 14518800 PMC2583061

[11] Michael YL , Berkman LF, Colditz GA, Kawachi I. Living arrangements, social integration, and change in functional health status. Am J Epidemiol. 2001;153(2):123–131. 10.1093/aje/153.2.123 11159156

[12] Ingersoll-Dayton B , Neal MB, Jung-Hwa HA, Hammer LB. Redressing inequity in parent care among siblings. J Marriage Fam. 2003;65(1):201–212. 10.1111/j.1741-3737.2003.00201.x

[13] Shields CG. Family interaction and caregivers of Alzheimer’s disease patients: correlates of depression. Fam Process. 1992;31(1):19–33. 10.1111/j.1545-5300.1992.00019.x 1559593

[14] Wenger GC. Support networks and dementia. Int J Geriatr Psychiatry. 1994;9(3):181–194. 10.1002/gps.930090303

[15] Lieberman MA , Fisher L. The effects of family conflict resolution and decision making on the provision of help for an elder with Alzheimer’s disease. Gerontologist. 1999;39(2):159–166. 10.1093/geront/39.2.159 10224712

[16] Brody EM , Hoffman C, Kleban MH, Schoonover CB. Caregiving daughters and their local siblings: perceptions, strains, and interactions. Gerontologist. 1989;29(4):529–538. 10.1093/geront/29.4.529 2521114

[17] Ashida S , Marcum CS, Koehly LM. Unmet expectations in Alzheimer’s family caregiving: interactional characteristics associated with perceived under-contribution. Gerontologist. 2018;58(2):e46–e55. 10.1093/geront/gnx141 28961867 PMC5946853

[18] Neufeld A , Harrison MJ. Unfulfilled expectations and negative interactions: nonsupport in the relationships of women caregivers. J Adv Nurs. 2003;41(4):323–331. 10.1046/j.1365-2648.2003.02530.x 12581097

[19] Özgül E , Akyol MA, Akpınar Söylemez B, Küçükgüçlü O. Caregiving self-efficacy in family caregivers of people with dementia: the role of knowledge of dementia and perceived social support. J Community Health Nurs. 2023;40(4):289–297. 10.1080/07370016.2023.2241454 37522835

[20] Shiba K , Kondo N, Kondo K. Informal and formal social support and caregiver burden: the AGES Caregiver Survey. J Epidemiol. 2016;26(12):622–628. 10.2188/jea.JE20150263 27180934 PMC5121430

[21] Singer B , Ryff CD, Carr D, Magee WJ. 1. Linking life histories and mental health: a person-centered strategy. Sociol Methodol. 1998;28(1):1–51. 10.1111/0081-1750.00041

[22] Nnoaham KE , Cann KF. Can cluster analyses of linked healthcare data identify unique population segments in a general practice-registered population? BMC Public Health 2020;20(1):798. 10.1186/s12889-020-08930-z 32460753 PMC7254635

[23] Chow SKY , Wong LTW, Chan YK, Chung TY. The impact and importance of clinical learning experience in supporting nursing students in end-of-life care: cluster analysis. Nurse Educ Pract. 2014;14(5):532–537. 10.1016/j.nepr.2014.05.006 24916407

[24] Pruinelli L , Simon GJ, Monsen KA, et al. A holistic clustering methodology for liver transplantation survival. Nurs Res. 2018;67(4):331–340. 10.1097/NNR.0000000000000289 29877986 PMC6023761

[25] Ahlqvist E , Storm P, Käräjämäki A, et al. Novel subgroups of adult-onset diabetes and their association with outcomes: a data-driven cluster analysis of six variables. Lancet Diabetes Endocrinol. 2018;6(5):361–369. 10.1016/S2213-8587(18)30051-2 29503172

[26] Thies W , Bleiler L. 2013 Alzheimer’s disease facts and figures. Alzheimers Dement. 2013;9(2):208–245. 10.1016/j.jalz.2013.02.003 23507120

[27] Institute of Medicine (US) Committee on the Future Health Care Workforce for Older Americans. Retooling for an Aging America: Building the Health Care Workforce. National Academies Press (US); 2008. 10.17226/12089 25009893

[28] Pinquart M , Sorensen S. Correlates of physical health of informal caregivers: a meta-analysis. J Gerontol B Psychol Sci Soc Sci. 2007;62(2):P126–P137. 10.1093/geronb/62.2.p126 17379673

[29] Christakis NA , Allison PD. Mortality after the hospitalization of a spouse. N Engl J Med. 2006;354(7):719–730. 10.1056/NEJMsa050196 16481639

[30] CDC. Assuring Healthy Caregivers, a Public Health Approach to Translating Research into Practice: The RE-AIM Framework. Kimberly-Clark Corporation; 2008.

[31] Chang Y-P , Seo Y, Von Visger T. Symptom experience among family caregivers: symptom cluster analysis. Innov. Aging. 2020;4(Suppl_1):152–153. 10.1093/geroni/igaa057.497

[32] Tarlow BJ , Wisniewski SR, Belle SH, Rubert M, Ory MG, Gallagher-Thompson D. Positive aspects of caregiving: contributions of the REACH Project to the Development of New Measures for Alzheimer’s Caregiving. Res Aging. 2004;26(4):429–453. 10.1177/0164027504264493

[33] Henriksson A , Hudson P, Ohlen J, et al. Use of the preparedness for caregiving scale in palliative care: a Rasch Evaluation Study. J Pain Symptom Manag. 2015;50(4):533–541. 10.1016/j.jpainsymman.2015.04.012 26004399

[34] Fortinsky RH , Kercher K, Burant CJ. Measurement and correlates of family caregiver self-efficacy for managing dementia. Aging Mental Health. 2002;6(2):153–160. 10.1080/13607860220126763 12028884

[35] Sorensen S , Pinquart M. Developing a measure of older adults’ preparation for future care needs. Int J Aging Hum Dev. 2001;53(2):137–165. 10.2190/1R0D-30TC-F4K1-F0DW 11758723

[36] Burgio LD , Collins IB, Schmid B, Wharton T, McCallum D, Decoster J. Translating the REACH caregiver intervention for use by area agency on aging personnel: the REACH OUT program. Gerontologist. 2009;49(1):103–116. 10.1093/geront/gnp012 19363008 PMC3695600

[37] Spitzer RL , Kroenke K, Williams JB, Löwe B. A brief measure for assessing generalized anxiety disorder: the GAD-7. Arch Intern Med. 2006;166(10):1092–1097. 10.1001/archinte.166.10.1092 16717171

[38] Hart LG , Larson EH, Lishner DM. Rural definitions for health policy and research. Am J Public Health. 2005;95(7):1149–1155. 10.2105/AJPH.2004.042432 15983270 PMC1449333

[39] Sclan SG , Reisberg B. Functional assessment staging (FAST) in Alzheimer’s disease: reliability, validity, and ordinality. Int Psychogeriatr. 1992;4(Suppl 1):55–69. 10.1017/s1041610292001157 1504288

[40] Hastie T , Tibshirani RJ, Friedman J. The Elements of Statistical Learning: Data Mining, Inference, and Prediction. 2nd ed. Springer; 2009. 10.1007/978-0-387-84858-7

[41] Bonikowski B , DiMaggio P. Mapping culture with latent class analysis: a response to Eger and Hjerm. Nat National. 2022;28(1):353–365. 10.1111/nana.12756

[42] Milligan GW , Cooper MC. An examination of procedures for determining the number of clusters in a data set. Psychometrika 1985;50(2):159–179. 10.1007/bf02294245

[43] Gaugler JE , Zarit SH, Pearlin LI. The onset of dementia caregiving and its longitudinal implications. Psychol Aging. 2003;18(2):171–180. 10.1037/0882-7974.18.2.171 12825767

[44] Xu L , Liu Y, He H, Fields NL, Ivey DL, Kan C. Caregiving intensity and caregiver burden among caregivers of people with dementia: the moderating roles of social support. Arch Gerontol Geriatr. 2021;94:104334. 10.1016/j.archger.2020.104334 33516077

[45] Tuttle D , Griffiths J, Kaunnil A. Predictors of caregiver burden in caregivers of older people with physical disabilities in a rural community. PLoS One. 2022;17(11):e0277177. 10.1371/journal.pone.0277177 36331949 PMC9635719

[46] O’Caoimh R , Calnan M, Dhar A, Molloy DW. Prevalence and predictors of caregiver burden in a memory clinic population. J Alzheimers Dis Rep. 2021;5(1):739–747. 10.3233/ADR-201003 34755048 PMC8543373

[47] Connors MH , Seeher K, Teixeira-Pinto A, Woodward M, Ames D, Brodaty H. Dementia and caregiver burden: a three-year longitudinal study. Int J Geriatr Psychiatry. 2020;35(2):250–258. 10.1002/gps.5244 31821606

[48] Sun Y , Ji M, Leng M, Li X, Zhang X, Wang Z. Comparative efficacy of 11 non-pharmacological interventions on depression, anxiety, quality of life, and caregiver burden for informal caregivers of people with dementia: a systematic review and network meta-analysis. Int J Nurs Stud. 2022;129:104204. 10.1016/j.ijnurstu.2022.104204 35247788

[49] Valente TW. Network interventions. Science. 2012;337(6090):49–53. 10.1126/science.1217330 22767921

[50] MetLife. The MetLife Study of Caregiving Costs to Working Caregivers: Double Jeopardy for Baby Boomers Caring for Their Parents. The MetLife Mature Market Institute; 2011.

[51] Döring C , Lesot M-J, Kruse R. Data analysis with fuzzy clustering methods. Comput Stat Data Anal 2006;51(1):192–214. 10.1016/j.csda.2006.04.030

