# Supplementary material for: Using Clustering Methods to Map the Experience Profiles of Dementia Caregivers
Source: Innov Aging. 2024 May 18;8(6):igae046. doi: 10.1093/geroni/igae046 (PMC11163925; doi:10.1093/geroni/igae046)
Supplement: igae046_suppl_Supplementary_Materials [file igae046_suppl_supplementary_materials.docx]

**Supplemental Material**

**S1. CONSORT Diagram**

**
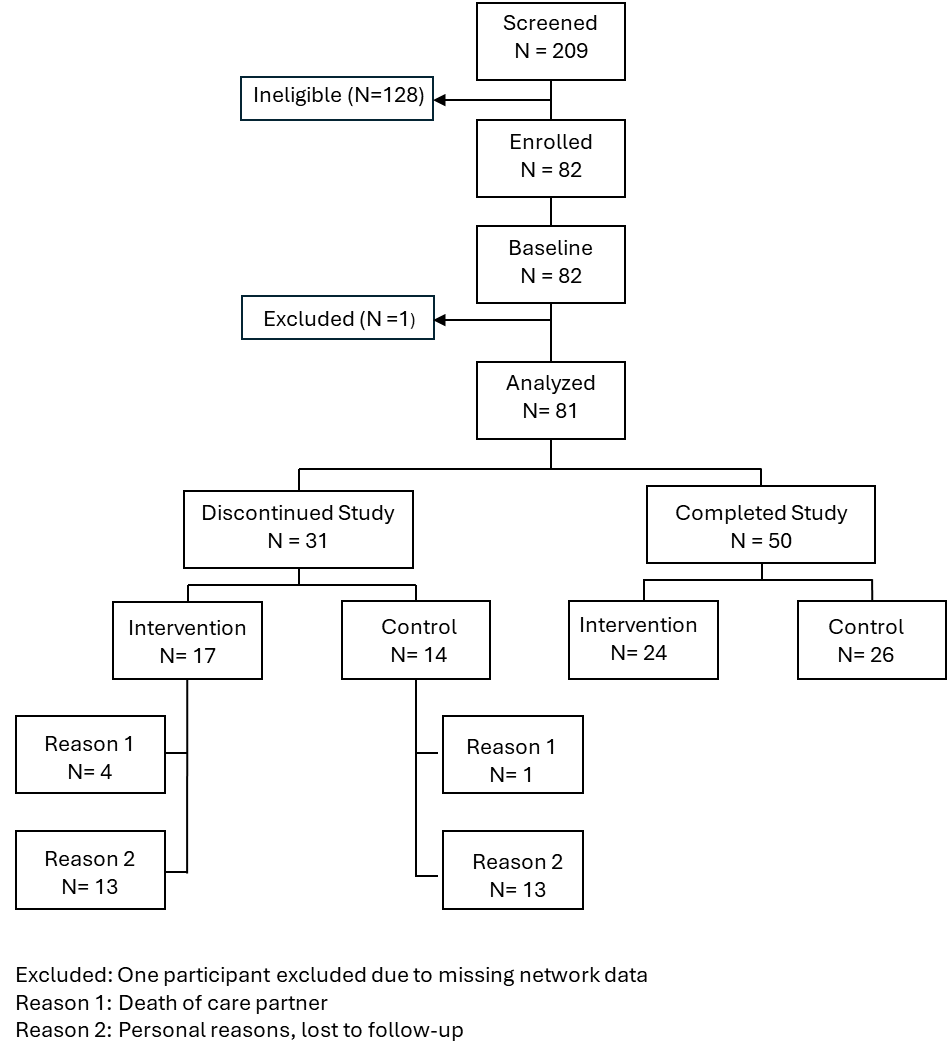
**

**S2. DESCRIPTION OF MEASURES**

**Positive Aspects of Caregiving.**^1^ **—***Some people say that, despite all the difficulties involved in helping someone with memory or health problems, good things have come out of their experience too. Below are a few of the good things reported by some people. Please rate how much you agree or disagree with these statements.*

1 = Disagree a lot; 2 = Disagree a little; 3 = Neither agree nor disagree; 4 = Agree a little; 5 = Agree a lot

Providing care and support to this individual…

1. made me feel more useful
2. made me feel good about myself
3. made me feel needed
4. made me feel appreciated
5. made me feel important
6. made me feel strong and confident
7. enabled me to appreciate life more
8. enabled me to develop a more positive attitude towards life
9. strengthened my relationships with others
10. strengthened my relationship with this individual

**Preparedness**^2^ **and Confidence.**^3^ **—**This measure draws from two sets of survey items. Note that responses to the second question, which is based on a 0-10 scale, were transformed to 0-4 prior to taking the mean of all 12 items.

*How well prepared do you think you are…*

0 = Not at all prepared; 1 = Somewhat prepared; 2 = Prepared; 3 = Well Prepared; 4 = Very well prepared

1. to take care of this individual’s physical needs?
2. to take care of this individual’s emotional needs?
3. to find out about and set up services for this individual?
4. for the stress of caregiving?
5. to make caregiving activities pleasant for both you and this individual?
6. to respond to and handle emergencies that involve this individual?
7. to get the help and information you need from the health care system?
8. Overall, how well prepared do you think you are to care for this individual?

*Now, I would like to ask you questions about how you feel and think about caring for the individual who was recently diagnosed with Alzheimer’s disease or dementia. How certain are you that you can…*

0 = Not at all certain to 10 = Very certain

1. get answers to all questions about services in your community
2. find organizations or agencies that provide services you need
3. arrange for services yourself
4. get answers to all your questions about this individual’s care

**Knowledge About Services for Older Adults.**^4^ **—***Please state how true each statement is for you in terms of how much you know about services for older adults.*

1 = Not at all true; 2 = Not really true; 3 = Neither true nor untrue; 4 = Somewhat true; 5 = Completely true

1. I am well-informed about the federal programs for older adults, including social security, Medicare, and Medicaid.
2. I know what I have to do to get help from the home health care agencies.
3. I know what I would need to do to get into a nursing home.
4. I am aware of how a stay in a nursing home or getting services from home health care are paid for.
5. I know where I can get information about different options for getting care.
6. I know which home health care agencies are active in my area.
7. I know which assisted living or nursing facilities are available in my area.

**Network Uplift.**^5^ **—**Participants in the baseline survey were asked to name people (or “contacts”) in their lives who “play important roles” in their caregiving context, including immediate family members and more generally, people who are important to the caregiver. To note, participants were also asked to nominate and evaluate themselves as people who play important caregiving roles. A total of 1,012 individuals were named, 931 (92.0%) of which were true network contacts and 81 (8.0%) of which were self-nominations. Information related to the self-nominations were excluded from analysis.

Later in the survey, participants were asked to identify (1=Yes, 0=No) specific contacts from their total list of contacts who offer different forms of instrumental and emotional support. The exact wording of the seven network support items is provided below. We next summed these seven items to produce a contact-level support score. For example, a contact with a score of seven is thus maximally supportive whereas a contact not nominated for any kind of support would have a score of 0. We then calculated a network uplift score for each participant, which corresponds to the sum of contact support per each participant’s total list of contacts.

[Name Generator Question] *Now, I want to ask you about people who may play important roles in helping you and this individual who received a diagnosis of Alzheimer’s disease or other dementia. To be complete, please list the names of people who fit the following descriptions.* *We would like to call these individuals the support network members.*

*1. First, please list the relationship or nickname of immediate family members of this individual such as his/her spouse and first-degree relatives including step and adopted children and siblings (i.e. their older sister will be J because that’s what her name starts with). Please make sure to include yourself if you are a part of this list.*

*2. Next, in addition to immediate family, please list the relationship or nickname of other persons who are important to this individual. This can include family, friends, and health or service providers…*

*3. Now, we are interested in people who are important to you. Please list the relationship or nickname of any other persons (family or non-family) who are important to you. We are particularly interested in those persons who provide emotional support, help you with everyday tasks, or give you helpful advice.*

[Name Interpreter Question] *Please answer the following questions by indicating appropriate network members for each question*.*^[[1]](#footnote-1)^

1 = Nominated by participant; 0 = Not Nominated

2. Who helps you provide care and/or support to this individual?

4. Who spends enough time helping this individual?

6. Who shows appreciation for your effort in helping this individual?

8. Who calls/emails you enough?

15. Who is very patient with this individual?

18. Who understands what you are going through with helping this individual?

20. Who provides enough help to you and others who help this individual?

**Burden.**^6^ **—***Please indicate how often you feel the following about helping this individual recently diagnosed with Alzheimer’s or dementia. How often do you feel…*

1 = Never; 2 = Rarely; 3 = Sometimes; 4 = Frequently; 5 = Nearly always

1. that because of the time you spend with this individual that you don’t have enough time for yourself?
2. stressed about balancing caring for this individual and trying to meet other responsibilities (work/family)?
3. angry when you are around this individual?
4. that this individual’s situation currently affects your relationship with family members or friends in a negative way?
5. strained when you are around this individual?
6. that your health has suffered because of your involvement with this individual?
7. that you don’t have as much privacy as you would like because you are caring for this individual?
8. that your social life has suffered because you are caring for this individual?
9. that you have lost control of your life due to this individual’s illness?
10. uncertain about what to do about this individual?
11. you should be doing more for this individual?
12. you could do a better job in caring for this individual?

**General Anxiety.**^7^ **—** *Over the last two weeks, how often have you been bothered by any of the following problems?*

0 = Not at all; 1 = Several days; 2 = More than half the days; 3 = More than half the days

1. Feeling nervous, anxious, or on edge

2. Not being able to stop or control worrying

3. Worrying too much about different things

4. Trouble relaxing

5. Being so restless that it’s hard to sit still

6. Becoming easily annoyed or irritable

7. Feeling afraid something awful might happen

**Network Malfeasance.**^5^ **—** Network malfeasance refers to the extent to which participants directly report frustration or negative emotion with their named contacts. From a list of all network contacts, participants identified (1=Yes, 0=No) specific contacts who they associate with some form of relational pressure or tension via seven questions (see below for exact wording). Then, following the same procedure as the network uplift variable, we summed these seven items across all contacts to create a network-level malfeasance score for each participant.

*Please answer the following questions by indicating appropriate network members for each question.*

1 = Nominated by participant; 0 = Not Nominated

10. Who doesn’t agree with you about what this individual is able to do for himself/herself?

12. With whom do you become upset regarding helping this individual?

13. Who is critical of you about how you help this individual?

14. With whom are you critical about how he/she helps this individual?

16. Who gives you unwanted advice about how you help this individual?

17. Who lacks patience with this individual?

19. Who becomes angry or upset with you regarding providing help this individual?

**Network Nonfeasance.**^5^ **—** Network nonfeasance refers to the extent to which participants feel that their contacts are not engaged enough in the caregiving context. From a list of all network contacts, participants identified (1=Yes, 0=No) specific contacts who they associate with some form of nonfeasance via four questions (see below for exact wording). Then, following the same procedure as the other network variables, we summed these four items across all contacts to create a network-level nonfeasance score for each participant.

*Please answer the following questions by indicating appropriate network members for each question.*

1 = Nominated by participant; 0 = Not Nominated

3. Who doesn't spend enough time helping this individual?

5. Who doesn't provide enough help to you or others who help this individual?

7. Who doesn't show appreciation for your effort in helping this individual?

9. Who doesn't call/email you enough?

**S3. ATTRITION**

When using a logit model and test if burden alone predicts the odds of dropping out, the coefficient for burden is statistically zero (*p*=.57). The same is true for positive aspects (*p*=.24), preparedness and confidence (*p*=.65), knowledge (*p*=.19), network uplift (*p*=.17), burden (*p*=.24), anxiety (*p*=.85), network malfeasance (*p*=.27), and network nonfeasance (*p*=.70). In sum, no single predictor on which the clusters are based can predict attrition. In contrast, as reported in the main text, there are statistically significant group differences in attrition rates by experience profile.

**References:**

1. Tarlow BJ, Wisniewski SR, Belle SH, Rubert M, Ory MG, Gallagher-Thompson D. Positive Aspects of Caregiving:Contributions of the REACH Project to the Development of New Measures for Alzheimer’s Caregiving. *Research on Aging*. 2004;26(4):429-453. <https://doi.org/10.1177/0164027504264493>

2. Henriksson A, Hudson P, Ohlen J, et al. Use of the Preparedness for Caregiving Scale in Palliative Care: A Rasch Evaluation Study. *J Pain Symptom Manage*. Oct 2015;50(4):533-41. <https://doi.org/10.1016/j.jpainsymman.2015.04.012>

3. Fortinsky RH, Kercher K, Burant CJ. Measurement and correlates of family caregiver self-efficacy for managing dementia. *Aging & mental health*. May 2002;6(2):153-60. <https://doi.org/10.1080/13607860220126763>

4. Sorensen S, Pinquart M. Developing a measure of older adults' preparation for future care needs. *Int J Aging Hum Dev*. 2001;53(2):137-65. <https://doi.org/10.2190/1R0D-30TC-F4K1-F0DW>

5. Ashida S, Marcum CS, Koehly LM. Unmet Expectations in Alzheimer's Family Caregiving: Interactional Characteristics Associated With Perceived Under-Contribution. *Gerontologist*. Mar 19 2018;58(2):e46-e55. <https://doi.org/10.1093/geront/gnx141>

6. Burgio LD, Collins IB, Schmid B, Wharton T, McCallum D, Decoster J. Translating the REACH caregiver intervention for use by area agency on aging personnel: the REACH OUT program. *Gerontologist*. Feb 2009;49(1):103-16. <https://doi.org/10.1093/geront/gnp012>

7. Spitzer RL, Kroenke K, Williams JB, Löwe B. A brief measure for assessing generalized anxiety disorder: the GAD-7. *Archives of internal medicine*. May 22 2006;166(10):1092-7. <https://doi.org/10.1001/archinte.166.10.1092>

1. * Participants were asked a total of 26 questions. Question numbers below for uplift, malfeasance, and nonfeasance refer to the order in which they appear in the battery of 26. [↑](#footnote-ref-1)
